# The influence of day care centres designed for people with dementia on family caregivers – a qualitative study

**DOI:** 10.1186/s12877-016-0403-2

**Published:** 2017-01-05

**Authors:** Signe Tretteteig, Solfrid Vatne, Anne Marie Mork Rokstad

**Affiliations:** 1 Norwegian National Advisory Unit on Ageing and Health, Vestfold Hospital Trust, Tønsberg, Norway; 2 Molde University College, Molde, Norway

**Keywords:** Dementia, Family caregiver, Day care centres, Respite, Support

## Abstract

**Background:**

Dementia is one of the most challenging age-related illnesses for family caregivers, whose care-related burden is well known. Research indicates that day care centres (DCCs) can reduce the caregiver burden and help family caregivers to cope with demands; however, the current body of knowledge is still tentative and inconsistent, and more research is recommended. The aim of this study is to provide an extended understanding of the situation of family caregivers and to examine to what extent DCCs can meet their need for support and respite.

**Methods:**

This study has a qualitative descriptive design using in-depth interviews with 17 family caregivers of people with dementia attending DCCs. The data analysis was undertaken using systematic text condensation.

**Results:**

Caregivers experience a complex role, with added responsibilities, new tasks, and emotional and relational challenges that are expressed through distressing emotions and demands for interaction. Additionally, the caregiving role leads to positive experiences, such as acceptance and adaptation, support and help, and positive changes in the relationship. Day care relieves family caregivers by meeting the person with dementia’s needs for social community, nutrition, physical activity, and structure and variety in everyday life. Using a DCC led to a higher quality of time spent together and easier cooperation, but it also produced some hard feelings and challenging situations. DCCs gave the caregivers a feeling of freedom and increased the time available to be spent on their own needs, to be social and to work or do practical tasks undisturbed.

**Conclusions:**

DCCs for people with dementia can give family caregivers support and relief and have a positive impact on the relationship between the family caregiver and the person with dementia. A more individualized program, in addition to flexible opening hours, would make DCCs even more effective as a respite service, positively influencing the family caregiver’s motivation and ability to care and postponing the need for nursing home placement.

## Background

Dementia is one of the most challenging age-related illnesses, not only for those who have been diagnosed with dementia but also for their family caregivers and healthcare professionals [1]. During the course of dementia, the need for assistance in the activities of daily living (ADL) increases, and the burden of continuous care and an extensive need for support falls on both family caregivers and social and health care service providers [2]. In the last decade, there has been a trend towards increased attention to day care facilities as an important part of community services [3]. Day care centres (DCC) offer both an activity programme for the service users and a respite service for the family caregivers [4–7]. The term ‘respite care’ is used to cover a range of services that can occur in the home [8], in a DCC, or in a residential setting [9].

The caregiver’s role and burden is well known and includes physical, psychological, social, and financial aspects [10, 11]. The term ‘caregiver burden’ is often used to describe this phenomenon, and it can be defined as “the degree to which a carer’s emotional or physical health, social life or financial status have suffered as a result of caring for their relative”[12]. Caregiver burden increases the risk of depression and anxiety disorder [13–17], and informal caregivers of people with dementia living at home experience care as more burdensome compared to informal caregivers of recently institutionalized people with dementia [18].

The caregiver burden can be associated with two main dimensions, the characteristics of the patient and the characteristics of the caregiver. *Patient characteristics* include the behavioural or psychological, disease-related, and socio-demographic factors related to the patient [19]. The *caregiver’s characteristics* (socio-demographic and psychological factors) influence their experience of caregiving. Female gender and cohabitation with the patient are associated with a larger burden, as are poor psychological health and poor religious coping skills [19].

Even though the majority of research has focused on burden and other negative aspects of family caregiving, positive aspects have been presented [20, 21], including a sense of meaning, a sense of self-efficacy, satisfaction, a feeling of accomplishment, and improved wellbeing and quality of relationships [22]. These positive experiences can help sustain family members in their work as caregivers [21].

DCCs providing a respite and support service have the potential to give family caregivers relief, reduce caregiver burden, and increase their motivation for their role as a caregiver [3, 7, 23]. These benefits can be summarized under four headings. 1) DCCs *facilitate separation time*, thereby giving family caregivers time that can be used for undisturbed work [8, 24, 25], rest, or other pursuits [6, 9, 25]. 2) DCCs seem to *reduce behavioural problems and the need for assistance with ADL*, but research on these effects is largely undocumented and tentative [6, 26, 27]. 3) *DCCs may reduce care demands, stress, and depression as well as increase wellbeing* [9, 28–30], but the results from previous research conflicts with this [5, 27]. Some studies indicate decreased symptoms of depression in caregivers when the person with dementia attends a DCC [9, 28], but other studies find no significant effect on wellbeing [25, 27]. 4) DCCs increase *motivation for care and postponement of the need for residential care* as they offer information and support regarding dementia-related topics, with the intention to reduce care-related stress [5, 8, 11, 25, 28, 31, 32]. Support for family caregivers aims to develop knowledge and skills in dementia care and prevent the risk of early institutional placement [11, 33].

Previous research indicates that DCCs can reduce the caregiver burden [34] and help family caregivers to cope with demands [5, 8, 27, 28, 35, 36]; however, current knowledge is tentative and inconsistent, and more research is recommended [7]. The aim of our study is to provide an extended understanding of the situation of the family caregiver and examine to what extent DCCs can meet their need for support and respite.

## Methods

This study has a qualitative descriptive design [37, 38]. In-depth interviews with family caregivers of people with dementia attending a DCC were used to explore their situation and the influence of the DCC on their needs for support and respite. To attain an extended understanding of their situation, we searched for family caregivers reflecting a variety of genders, ages and relationships with the person with dementia. In the in-depth interviews, the participations were given the opportunity to share detailed descriptions of their everyday life experiences, giving the researcher access to the world of their life [39]. These descriptions of lived experience can provide us with an extended understanding of the influence of DCCs on the family caregivers’ experience of relief and support.

The participants were recruited from the research project ‘*Effects and costs of a day care centre programme designed for people with dementia – a 24 month controlled study’* (ECOD) [40]. The ECOD study has a quasi-experimental design including a group of day care users and their closest family caregivers (intervention group) and a comparison group of people with dementia, with no access to day care, and their caregivers. The ECOD study is funded by unrestricted grants from the Research Council of Norway and has been accepted by the Regional Committee in Ethics in Medical Research of South-East Norway.

The criteria for the inclusion of participants in the present study were that they are family caregivers to a person with dementia attending a DCC designed for people with dementia and, furthermore, that they have face-to-face contact with this person a minimum of once a week. Additionally, the sample of participants was chosen to represent both genders, a variety in age and different relationships to the person with dementia (spouses, children/children-in-law, living together with the person or not). There were no exclusion criteria. The participants gave written, informed consent to take part in the study at the time they were recruited.

Seventeen individual semi-structured interviews were carried out in March and April 2015. A stratified sample for qualitative interviews based on gender, age, relationship to the person with dementia, and cohabitation was chosen from the participants in the ECOD study (see Table 1). The variations in the characteristics of the participants represented different needs and settings, and thus, there was diversity in the data collected. The participants were connected to six DCCs located in rural districts and cities.

**Table 1:** Characteristics of the participants and the persons with dementia

Family caregiver’s role	Family caregiver’s age	Living together with the person with dementia	Working	Person with dementia’s role	Severity of dementia (CDR^a^)	Person with dementia’s age
1. Son	52	No	No	Father	Very mild	74
2. Daughter	56	No	No	Mother	Mild	77
3. Wife	74	Yes	No	Husband	Mild	77
4. Daughter	59	No	No	Mother	Very mild	80
5. Daughter-in-law	47	No	No	Mother-in-law	Mild	82
6. Son	47	No	Yes	Mother	Mild	80
7. Wife	79	Yes	No	Husband	Moderate	83
8. Son	46	No	Yes	Mother	Very mild	76
9. Wife	77	Yes	No	Husband	Mild	87
10. Wife	72	Yes	No	Husband	Moderate	81
11. Wife	86	Yes	No	Husband	Mild	92
12. Daughter	58	No	No	Mother	Mild	81
13. Husband	76	Yes	No	Wife	Mild	74
14. Daughter	70	No	No	Mother	Mild	96
15. Daughter	65	No	No	Mother	Moderate	87
16. Husband	77	Yes	No	Wife	Mild	72
17. Wife	74	Yes	No	Husband	Mild	79

^a^ *CDR* Clinical Dementia Rating Scale

There were 17 family caregivers who participated in the study, and they were recruited by the leaders of the DCCs. These participants had attended a DCC with activities designed for persons with dementia for approximately 2 - 18 months, 2 - 5 days a week. The interviews were made face-to-face at the DCC (n = 4), by Skype (n = 2), or in the participant’s home (n = 11). The interviews lasted for 30 - 90 minutes.

Based on the aims of the study, research findings, and national political documents, the interview guide (Table 2) was arranged using open themes that invited the participants to describe their situation and experiences with the DCC.

**Table 2:** The interview guide

Themes	Sample question
1. Introduction - relationship	Please tell me about your situation after NN got dementia? In what way does the dementia disease affect your daily life? In what way does the dementia disease influence your relationship?
2. Day care - in the beginning	How did you experience the process ahead of DCC attendance and the first days and weeks?
3. A typical day	Can you describe a typical day when NN is attending the DCC (before, during and after)?
4. Day care as a support and respite service	To what extent and in what way do you experience the DCC to be a respite service for you as a family caregiver?
5. The content and quality of the DCCs	What are your experiences with the content and quality of the DCC? Are there some elements of the DCC service that are more important than others for you?
6. Summary questions	DCCs are considered to postpone the need for residential care. What do you think about that? What do you think about the future? In summary, what does the DCC represent for you?

The data analysis was performed using systematic text condensation [37, 38], according to the following four steps: 1) Total impression – from chaos to *themes* **.** The whole text was read through several times to get an overview of the total content and to identify the overall themes to be further analysed. Three themes describing how the family caregivers experienced their complex caring roles and four themes related to how the day care centre influenced this role were identified. 2) Identifying and sorting *meaning units* – from themes to codes, with the codes being a text fragment containing some information about the identified themes of interest. 3) Condensation – from code to meaning, where the data were reduced to a decontextualized selection of meaning units and sorted as *thematic code groups* across the individual participants. In this step, we went back to the transcript of interviews seeking meaningful quotations describing the content of the codes. The quotations demonstrate both similarities and differences in how the family caregivers experience their role and how the DCC influence theirs. Differences in role, gender and age are presented in the summary of results. Initially, the first author performed this process, while further discussions and reorganization occurred in collaboration with the co-authors. The Nvivo qualitative data analysis program was used. 4) Synthesizing the codes into descriptions and concepts [38].

## Results

Two main themes were identified: the family caregivers’ complex caring roles, and the influence of the DCC on the caregivers’ situations. The individual descriptions of their role as caregivers are important to understand the influence of the DCC service on their situation. The themes are presented with code groups and sub-code groups in Table 3, and they will be further described in the following sections.

**Table 3:** Main findings sorted as themes, code groups, and sub-code groups

Themes	Code groups	Sub code groups
The complex caring role of the family caregiver		
Added responsibilities and new tasks	Guiding and assistance in activities of daily living	Providing assistance in • practical tasks • personal hygiene Being sensitive to the persons’ psychological needs Adapting to changes in person’s social skills
Emotional and relational challenges	Distressing emotions	Feeling • guilty conscience • sorry for • being tied down
	Demanding interaction	Increased dependency Disagreements and misunderstandings Confrontations Nagging Lack of interests and engagement Adapt to the needs of the person with dementia
Resources affecting the situation positively	Acceptation and adaptation	Accept the situation Find new solutions Use humor With God’s help
	Support and help	Help from friends and family Seek knowledge in the literature Receive professional help
	Positive changes in the relationship	Doing things together Positive contact Positive feedback
The influence of the DCCs on the family caregiver situation		
Respite – assistance to meet the needs of the person with dementia	More fellowship	Inclusion Social support
	Meeting basic needs	Nutrition, sleep, and rest
	Improved structure in everyday life	Schedule of daily events
	More variation, activity and meaning	Physical activity Do something meaningful Get out of home
Positive and negative influence on the relationship	Higher quality of time spent together	Less nagging More calm, tired, and positively exhausted Something to talk about
	Easier to cooperate with	Increased wellbeing Increased engagement and level of function Fewer conflicts
	Hard feelings and situations	Tricking and lying
Increased separation time - more time to meet their own needs	Increased time to spend on own needs	Rest and relaxation Activities Activities Work undisturbed Practical activities Family and friends Feeling of freedom
Needs that are not met by the DCCs	Flexibility	Flexible and long opening hours and days
	Information, communication and information	Needs of • information about schedule and content of activities in the DCCs • feedback about the participant • information about dementia-related topics (was obtained in classes for family carers )
	Quality and content designed for people withdementia	Lack of tailored activities Lack of inclusion and social support

### The complex caring role of the family caregiver

#### Added responsibilities and new tasks

The burden related to the symptoms of dementia and the person with dementia’s need for help have a major impact on the family caregivers’ role, which is described as a complex caring role. How the disease influenced the person with dementia was related to cognitive, psychological, social, and behavioural changes. These changes increased the need for guidance, support, and practical assistance, which was mainly covered by the family caregivers:

*He stands still and wonders what to do. Then, I say to him: The cups are placed over there; you may pick up a couple of cups and then find the cutlery. Then, he stands there wondering again (wife, 11).*

A family caregiver’s daily life is full of such situations. Even if they receive public or private services at home, they still have to assist the person with dementia in organizing their day, remembering appointments, cleaning the house, taking care of the laundry, and so forth. To receive public service support in practical tasks every third week does not fulfil their needs – such as cleaning a fouled-up toilet. Family caregivers spend a lot of time and effort cleaning and washing, and some of them stated that assistance in these tasks gave them the best kind of respite:

*My feeling of respite is related to the fact that they clean her apartment, they vacuum the floor and change her bed linen (…). For me, that’s respite (daughter, 15).*

Some family caregivers stated that increased problems related to hygiene and bowel incontinence could be the main reason for the need of a nursing home placement.

*As long as the brain can tell her that she needs to go to the toilet, I think we will fix it. But if the brain doesn’t tell her, it will be a problem. I think everybody will understand that (…). Yes, it will be difficult (…), there will be a bad smell all over the place (son, 8).*

Psychological changes in the person with dementia, such as apathy, lack of inhibition, anger, and offensive comments, influenced the family caregivers, and they became less socially active than before, due to the person with dementia’s waning interest in their children and grandchildren. Additionally, the person with dementia could get irritated or angry for what the caregiver regarded as no apparent reason.

#### Emotional and relational challenges

Family caregivers described experiencing challenging emotions and demanding interactions. The caregivers felt sorry for the person with dementia because he or she had become dependent, and they struggled with a guilty conscience when they left them alone.

*Mostly, I can do the things I need to do when he is at home, but I have a guilty conscience for letting him sit in a chair when I do other things (wife, 17).*

This was mainly the case for spouses but also for some of the children. Some of the family caregivers experienced a lot of nagging and felt that they had to lie, which made them feel bad. For instance, some did not mention that they planned to visit family members living far away because they wanted to avoid a lot of worry and continuous telephone calls from the person with dementia before and during the visit. Both spouses and children/children-in-law felt that they were trapped in their caregiver role. Many of them stated that they could never relax and that they worried about what might occur when the person was alone. This was the case both for family caregivers living with the person and for those living separately.

*She isn’t the kind of person who goes out and disappears (…), but all the time you are on guard (husband, 13).*

Continuous monitoring of the person with dementia resulted in exhaustion, and some caregivers described a lack of sleep. Several stated that the person with dementia was confused and called them repeatedly around the clock. Additionally, some caregivers received accusations and had agonizing confrontations:

*He was very concerned about something that I had done wrong, it was something wrong with me all the time. I had stolen money or destroyed things (wife, 7).*

Being wrongfully accused or repeatedly involved in confrontations was an especially sensitive experience for the spouses. Some of them cried when they were talking about this, and they described the experience as getting stuck in a role that they strongly disliked. That the person with dementia took less of an interest in daily life was also described as a burden:

*If I comment on something on the TV or in the newspaper and he is not interested, he seldom responds to me (wife, 11).*

The fact that the person with dementia no longer showed interest in matters of daily life was described as a loss, especially for the spouses.

#### Resources affecting the situation positively

Although the descriptions shared by the family caregivers contained mostly difficult situations, they also presented positive experiences as to how they accepted and adapted to the new situation, and they received support and help. An example of adaptation was a spouse who stopped driving and sold the car in solidarity with her husband, who did not understand that he had lost his driving license. By doing this, she avoided difficult confrontations.

Nevertheless, some family caregivers also experienced positive changes in their relationship, which were described as an increased emotional presence or thankfulness from the person with dementia:

*When I see how easily I can please her, I think, why don’t I do this more often? (daughter, 4)*

Some family caregivers stated that the person with dementia had become more tolerant, was seeking more contact, and was increasingly socially active. In one family, the contact between the father with dementia and the children was restored after many years without a relationship. Many described positive experiences in their interactions with public health services, which offered rapid help and high-quality support.

### The influence of the DCCs on the family caregiver situation

#### Respite – assistance to meet the needs of the person with dementia

Family caregivers experienced the DCC as a service that represented something safe and routine in the person with dementia’s daily life. They knew that the person was occupied with something meaningful, which gave them a break from the need to pay a visit on those days (children). The fact that they got themselves out of their home and were included in an organized fellowship was a relief for the caregivers. Without such a service, the person with dementia would have spent most of the day on their own, a situation that would reinforce the caregiver burden:

*For me, it feels so good to know that my mother-in-law is in a place where she enjoys herself. I know she is active and something is happening in her life from half past nine to half past two, Monday to Friday. (…). She gets exercise. It is this mix of mental and physical activities that increases her wellbeing. Otherwise, she would have been sitting at home watching television (daughter-in-law, 5).*

To meet the person’s nutritional needs was described as a challenge: if the person with dementia had a poor appetite, altered experience of taste, or offensive behaviour during the meal, then a situation commonly associated with pleasure and enjoyment became a burden. Therefore, mealtimes offering good food and fellowship were described as one of the most important activities at the DCCs.

The DCCs influenced the circadian rhythms of the people with dementia, regulating the structure of the day in a positive way; they had better sleep at night because they were more active and awake during the day:

*She is often tired when she is back from the day care centre, but she generally sleeps a lot. She often lies down and sleeps a lot; she is very tired (daughter, 14).*

Better sleep at night entailed better nights for the caregivers as well; spouses were less on alert, and children and children in-law received fewer telephone calls during the night.

The new situation, in which the person with dementia should be attending DCC, could be emotionally difficult for family caregivers, especially when they felt that they had to persuade or dupe the person to go to there. This was described especially as a challenge in the very first days or weeks and led to worries and feelings of guilt. It was therefore important that the person with dementia was motivated by the DCC’s activities, tailored day programmes and activities. Social support was reported to be helpful in this situation.

Physical activities were described as an important part of the DCC service. Some family caregivers, mainly spouses, often took responsibility to get the person with dementia outdoors to have some daily exercise. On the days when their spouses attended the DCC, they were released from this duty. Some of the persons with dementia associated the DCC with their previous work and with being a useful person. Many of them also had regular tasks to perform at the DCCs, and this increased their motivation to go there without their caregivers making too much of an effort.

#### Positive and negative influences on the relationship

Attendance at a DCC influenced the person with dementia positively, giving rise to better moods, less nagging, and more calmness. Confrontations were less common, and the individuals with dementia had more to talk about in conversations. Furthermore, their cooperation was better, and they shared more pleasant moments with each other:

*Mostly there is no rush when he is at day care. One telephone call maybe, but then we mostly have a pleasant chat or give each other short messages. It's not the same nagging about things as it was previously (son, 1).*

Some caregivers preferred to call their parents on the days of DCC attendance because they experienced a nicer chat on those days compared to the days when they stayed at home. Additionally, DCC attendance increased their engagement, practical functioning, and wellbeing. For instance, the person with dementia was often concerned about what to wear, and they got dressed up to go there.

S*he blossoms when she is there (…). Otherwise, she is not keen to dress up or change clothes (daughter, 2).*

#### Increased separation time - more time to meet their own needs

Only two of the caregivers, both children of people with dementia, were working. The rest of the participants were retired or out of work for other reasons. The two that were still working experienced the DCCs as a respite from their worries during work hours. The fact that their mothers received food and support while the family caregivers were at work was absolutely essential for the mothers to be able to stay at home. For those not working, day care gave more time for rest and relaxation, both for spouses and children:

*The day care means a lot. It gives me at least two days a week to do what I want. I can pay some attention to my own needs as I usually use all my time to attend to his needs (wife, 3).*

Many family caregivers used the separation time for activities and practical tasks at, or away, from home. They spent time with family and friends, and several of them expressed the sense of freedom they achieved when the person with dementia stayed at the DCC:

*When I wake up in the morning, I know that this day is mine. Today I can do things I cannot do the other days: be at home, together with grandchildren or with my daughters, or just be myself (daughter, 2).*

The description of this sense of freedom testifies to the strong commitment that many family caregivers experience.

#### Needs that are not met by the DCCs

Overall, the family caregivers were very satisfied with the DCCs. However, they described some needs the DCCs did not meet, such as the need for an extended number of opening days if they were going away on vacation and longer and more flexible opening hours in the evenings, at night, or during the weekends:

*I would have liked some days at the day care centres to be a little longer because if I, for instance, go out to have lunch with my nieces, they like to eat at 13.30, not at 12. As my husband comes home half past two, I am in a bit of a hurry, you see (spouse, 11).*

Family caregivers described difficulties during holidays and vacations due to closed DCCs. This situation caused a break in the daily routines, which could lead to increased confusion and more stress for the family. Additionally, it was not easy for family caregivers to go away for a vacation when the DCC was closed:

*Yes, it gives me relief. Absolutely! However, when the day care centre is closed for some days, or you want to go for a vacation or something like that, it is really difficult (daughter, 15).*

The need for information about dementia-related topics was mainly covered by courses for family caregivers, which were offered as a service in most municipalities but not organized by the DCC. However, some caregivers expressed the need for more direct feedback and information from the DCC staff about the schedules and the content of the days at the DCC. The memory problems arising from dementia make it difficult for the person with dementia to communicate and share experiences about what has occurred during the day. Hence, both children and spouses would like to have more information about the daily activities and schedules of the DCCs.

Some of the family caregivers reported a lack of individualized care and had the impression that the DCC staff failed to be inclusive and give social support to the people with dementia. The activities were also not adequately tailored to the interests and functional levels of the individuals with dementia:

*My opinion of the content and quality of the day care? Well, I don’t know what to say. I am sure it is OK for those who belong to this district (…) but she did not grow up here, she didn’t attend any of the schools in this area. So when they are driving along looking at these schools, it means nothing for her (daughter, 2).*

If the service fails to tailor the activities to the service users’ interests and needs, then the motivation to go there decreases. As a result, the person with dementia is dissatisfied, and the family caregivers feel bad because they have to increase their efforts to motivate the person to go to the DCC. This situation can add to the caregiver burden.

The DCCs gave the family caregivers a valuable break from the responsibility, the workload of practical tasks, and the feeling of being tied down. However, in addition, they stated that their own care and support were crucial in enabling the person with dementia to stay at home. If, for various reasons, they were not able to continue in the caregiving role, then the DCC as the only support would not be adequate to meet the person with dementia’s care-related needs.

## Discussion

Participants described many elements of their situation that resulted in a rich and powerful resource for understanding how DCCs influence their role as caregivers. The findings are presented by utilizing the major themes: (a) Respite and shared responsibility, (b) Day care attendance and the influence on the relationship between the family caregiver and the person with dementia, (c) Limited opening hours - consequences for the caregiver’s social life, (d) Quality through an individualized programme and cooperation with caregivers, and (e) Does DCC attendance postpone the need for nursing home placement?

### Respite and shared responsibility

Day care relieves family caregivers by meeting the person with dementia’s needs for social community, nutrition, physical activity, and structure and variety in everyday life. This experience of relief was independent of the relationship to the person (i.e., spouse or children), gender, and whether they lived in the same household. The results from previous research shows that day care provides family caregivers with a feeling of shared responsibility, in the sense that someone is able to take responsibility for the person with dementia if the family caregivers should become ill or die. If this happens, then the caregivers feel safe that the staff at the DCC know the person with dementia’s situation and condition and can give that information to other relevant health units in the municipality [7]. The present study shows that DCCs additionally provide the families with a sense of shared responsibility and relief while they are still active in their caregiving role.

The caregiver’s responsibility leads to a feeling of commitment. Previous research shows that female caregivers find themselves tied into the care situation to a larger extent than do men [41]. Our study shows that men and women and spouses and children all feel the need to be on their guard, adapting themselves to the needs of the person with dementia all the time, as it feels difficult to leave them alone. The DCC gives the relatives valuable respite from the experience of being tied down, but because of the limited opening hours and days, this service cannot fully meet the person with dementia’s needs if the relatives are unable to provide care for various reasons. In these situations, the person with dementia would need help from other family members, more frequently home-based services or residential care.

The increased need to handle practical tasks can be a physical burden for family caregivers, especially those in poor health. Many of these caregivers do all the cooking and cleaning, and they take care of the laundry. These tasks are to be taken care of in addition to, or instead of, receiving public or private practical assistance. Attendance at a DCC makes these daily responsibilities less demanding for family caregivers as they can carry out the necessary practical tasks without being disturbed. Bowel incontinence of the person with dementia was described as a great burden that could trigger the need for nursing home placement. We may assume that family caregivers would like to use more of the separation time to rest and take part in pleasant activities if they were relieved from the large amount of practical tasks with which they are often saddled. Individualized and tailored arrangements and flexibility in assistance with practical tasks at home can likely improve the caregiver’s situation.

This study revealed important information about the complexity of the family caregiver role according to the challenging relationship, new tasks and the added responsibility for meeting the needs of the person with dementia. Family caregivers felt that the DCC definitely gave respite and a feeling of shared responsibility for the caring tasks, although there were some limitations, which will be further discussed below.

### Influence of day care attendance on the relationship between the family caregiver and the person with dementia

The fact that the caregivers’ basic need for rest is met gives them new energy and more patience to handle relational challenges. The mental burden that comes with caring for a spouse with dementia can be associated with losing a sense of community with the partner [42], and this was confirmed by the participants in our study. Additionally, the study reveals that children also experience this kind of loss. However, day care provides new impulses and gives the people with dementia more to talk about. A person with dementia cannot always remember their experiences at day care, and so the caregivers ask for information about what is occurring at day care so that they can use it in conversations. Information from the staff shared in a notebook, by SMS, or mail can facilitate daily chats.

The family caregivers experienced fewer conflicts and less nagging when the person with dementia participated in the DCC. That change led to increased wellbeing and a higher quality of the relationship with the person with dementia. A good relationship increases the probability of a positive and meaningful experience in the role of caregiver. Knowledge about dementia can lead to a new understanding and more empathy for the person with dementia and hence increased acceptance of their caring role [43]. Maintaining or building a good relationship between the caregiver and the person with dementia might have a positive impact on the role of the caregiver. Previous research reveals that caregiving tasks that are experienced as meaningful can be a potential buffer against caregiver burden and influence the caregiver’s mental health positively [44]. The experience of a good relationship increases the likelihood of the caregiver valuing the person with dementia more and not focusing mainly on the problems. Furthermore, caregivers who experience a high degree of mutuality in the relation tolerate staying in the caregiving role longer than do caregivers who experience low levels of mutuality [45]. Thus, being a caregiver might fill a need for meaning in life, which can motivate and empower people to handle even the most difficult situations. According to Logo-therapy (Frankl, 1963), shifting the focus away from our own needs to concentrate on satisfying others is health-promoting and can make life more meaningful [46, 47]. The DCC’s contribution to a better relationship and a deeper understanding of the situation between the family caregivers and the person with dementia can strengthen the caregivers’ ability to care and reduce the caregiver burden.

### Limited opening hours - consequences for the caregiver’s social life

This study reveals that limited opening hours at the DCCs influence the caregiver’s potential to have a social life. Short and fixed opening hours and the lack of service during public holidays and vacations give relatives limited opportunities for an active social life, especially in the evenings. The need for flexible opening hours, as expressed by the family caregivers, confirms previous research that showed that flexible opening hours and programmes are important for the DCCs to provide respite [31, 36, 48]. Social stress is one of the factors affecting the burden on the family caregiver [10, 12]. Therefore, helping families to address their need for ‘social capital’ (contact that gives access to social, emotional, and practical support) has a positive impact on the caregiver burden [49]. To meet these needs, DCCs should be open during holidays and weekends, and opening hours should be longer and more flexible.

### Quality through an individualized programme and cooperation with caregivers

Some family caregivers reported that the DCC programme was not adjusted to the person's background and functional level and that the person with dementia felt uncomfortable. The caregivers felt guilty, and the situation increased their feeling of burden. Staff knowledge about the person’s identity and the possibility of individualized care had an influence on the family’s feeling of respite. Previous research describes that the quality and expertise of dementia care, shown by tailored day programmes and activities for the person with dementia, give the person social support and activities, which enhance coping [3–5].

In contrast to previous research showing the need for information and support for family caregivers to be offered by the DCC, the caregivers in our study received this type of information at classes for family caregivers; these classes were provided through a support and educational programme organized by the municipalities [50]. Those who attended these classes had lower expectations of education and support from the DCC than had emerged in previous research [7]. A few of the participants were offered individual support and structured meetings with the DCC staff, and they found this service very useful. In these meetings, caregivers received information about the DCC programme and individualized care, and this information made them confident of the quality of the service. Only a few caregivers had been offered individual meetings, but many of them expressed a need for this. As many families have limited or no daily contact with the DCC staff, individual meetings are important for cooperation, the exchange of information, and support. Additionally, such meetings gave the caregiver the opportunity to share important information with the staff about the person with dementia. Hence, regular meetings with family caregivers to exchange information and experiences should be given high priority.

### Does DCC attendance postpone the need for nursing home placement?

Some of the family caregivers stated that the DCC, combined with their own running care, surprisingly postponed the need for nursing home placement. It should be noted that a premise for this statement is the need for the family caregiver to be relatively healthy in addition to the functional level of the person with dementia, especially with relation to hygiene. Many of the family caregivers in the study were convinced that the person with dementia was unable to live on their own, even with the availability of more home-based public care. To postpone nursing home placement, daily support from family caregivers is crucial in addition to support from the DCCs.

### Summary of implications

To summarize the implications of the findings in this study, the following aspects are important. The family caregivers feel responsible for the person with dementia’s need for nutrition, physical activity and social stimulation. To share this responsibility with the DCC gives them relief that presupposed individual adaptation of the service. Therefore, meeting these needs should be a high priority in DCCs.

Day care seems to have a positive influence on the relationship between family caregivers and the person with dementia; it provides family caregivers with more energy and patience, reduces behavioural challenges and provides the family caregiver and the person with dementia with positive topics of conversation. Information about content and schedules at the DCC will help family caregivers in their daily conversation with the person with dementia.

According to this study, DCCs might contribute to the postponement of nursing home placement. However, this result depends on flexible opening hours, high quality of the DCC programme and regular cooperation with family caregivers.

### Methodological considerations

The aim of this study was to provide an enhanced understanding of the impact on family caregivers of DCCs designed for persons with dementia. The in-depth semi-structured interviews provided a rich source of material with personal descriptions related to the aim of this study. The participants represented a diversity of genders, ages, and caregiver roles (spouses, children, living together or separately). Moreover, the sample represented caregivers of people with various degrees of dementia and with different numbers of hours and days at the DCCs, which is a strength of the current study. There were only two participants still working (both sons), which resulted in limited information about job-related issues. There are also other limitations of this study. Only one interview was made with each participant, and hence, there was no possibility to study the researchers’ interpretations of the data or for the participants to add further information. The data were originally collected to explore the influence of DCC on family caregivers and not to focus on describing their situation generally. Other limitations due to sampling techniques may influence the external validity, and, because of this, the results cannot be generalized to other groups of caregivers. However, the results can elucidate the needs of similar groups of family caregivers, their situations, and how their needs for support and respite can be met.

## Conclusion

The current study supports findings from previous studies describing caregiver burden and the need for caregiver support. Our findings add an extended understanding to how DCCs designed for persons with dementia can offer relief and support for family caregivers and increase their ability to meet the needs of the person with dementia on a day-to-day basis. This study reveals a possible positive impact of DCCs on the relationship between the family caregiver and the person with dementia and the possibility to postpone the need for nursing home placement.

Future research should focus on how the person with dementia experiences day care attendance and further explore how DCCs influence the relationship between the person with dementia and their family caregiver.

## Abbreviations

DCC: Day Care Centres
